# Reply to: Temporal evolution of tomographic findings of pulmonary infection in COVID-19

**DOI:** 10.31744/einstein_journal/2020CE6040

**Published:** 2020-10-02

**Authors:** Marcela Emer Egypto Rosa, Marina Justi Rosa de Matos, Renata Silveira Olimpio de Paula Furtado, Vanessa Mizubuti Brito, Lucas Tadashi Wada Amaral, Gabriel Laverdi Beraldo, Eduardo Kaiser Ururahy Nunes Fonseca, Rodrigo Caruso Chate, Rodrigo Bastos Duarte Passos, Gustavo Borges da Silva Teles, Murilo Marques Almeida Silva, Patrícia Yokoo, Elaine Yanata, Hamilton Shoji, Gilberto Szarf, Marcelo Buarque de Gusmão Funari

**Affiliations:** 1 Hospital Israelita Albert Einstein São PauloSP Brazil Hospital Israelita Albert Einstein, São Paulo, SP, Brazil.

Dear authors,

We thank you for your kind comments and agree upon the existing doubts about the real meaning of changes in images of the coronavirus disease (COVID-19) in the long term. Some authors bring an extrapolated reflection of other infections, including those related to outbreaks caused by other coronaviruses,^(1)^ and indicate a potential persistence of some changes in images, including with repercussions in pulmonary function tests more than 10 years after the infectious insult.

However, the great case presented by the authors^(2)^ showed that even some changes usually related to fibrosis can regress and, eventually, disappear. We have also observed some extensive lesions, sometimes with similar characteristics to those reported by the authors, which presented significant improvement during evolution. On the other hand, we have seen ground-glass opacities persisting for a long time in some patients. Although the progressive imaging behavior in COVID-19 is relatively predictable,^(3,4)^ there are still cases that challenge this pattern.

In addition, other conditions sometimes overlap the infection caused by the severe acute respiratory syndrome coronavirus 2 (SARS-CoV-2). Besides greater incidence of venous and arterial thromboses^(5)^ described in these patients, we have also observed coinfection and superinfection, which are sometimes dramatic cases, generating imaging patterns that challenge the most classic aspects, as illustrated by us,^(3)^ and also by other Brazilian authors.^(4)^ Yet, we believe it is worth mentioning the higher incidence of barotrauma in these patients,^(6)^ another factor that may contribute to pulmonary damage. All these complications can also influence in the progressive imaging changes of COVID-19 and have the potential to leave residual findings in the parenchyma.

Therefore, we consider appropriate that COVID-19 patients must maintain clinical follow-up by means of functional tests and eventual tomographic controls, particularly those with more severe and extensive cases and presenting complications in the course of the disease. This is an evolving subject, with vast potential for researches.
