# Supplementary material for: Reply to: Temporal evolution of tomographic findings of pulmonary infection in COVID-19
Source: Einstein (Sao Paulo). 2020 Oct 2;18:eCE6040. doi: 10.31744/einstein_journal/2020CE6040 (PMC7531896; doi:10.31744/einstein_journal/2020CE6040)
Supplement: Translation

# Translation: Resposta para: Evolução temporal dos achados tomográficos da infecção pulmonar na COVID-19

**Authors:** Marcela Emer Egypto Rosa, Marina Justi Rosa de Matos, Renata Silveira Olimpio de Paula Furtado, Vanessa Mizubuti Brito, Lucas Tadashi Wada Amaral, Gabriel Laverdi Beraldo, Eduardo Kaiser Ururahy Nunes Fonseca, Rodrigo Caruso Chate, Rodrigo Bastos Duarte Passos, Gustavo Borges da Silva Teles, Murilo Marques Almeida Silva, Patrícia Yokoo, Elaine Yanata, Hamilton Shoji, Gilberto Szarf, Marcelo Buarque de Gusmão Funari

**Affiliations:** 1 Brasil Hospital Israelita Albert Einstein, São Paulo, SP, Brasil.

Caros senhores autores,

Agradecemos pelos gentis comentários e concordamos sobre as dúvidas existentes quanto ao real significado das alterações por imagem da doença pelo coronavírus (COVID-19) em longo prazo. Alguns autores trazem uma reflexão extrapolada de outras infecções, inclusive aquelas relacionadas aos surtos causados por outros coronavírus,^(1)^ e apontam para uma potencial persistência de algumas nas imagens, inclusive com repercussão em provas de função pulmonar mais de 10 anos após o insulto infeccioso.

O belo caso apresentado pelos autores^(2)^ mostra, entretanto, que mesmo algumas alterações geralmente relacionadas à fibrose podem regredir e, eventualmente, desaparecer. Também temos observado casos de lesões extensas, por vezes com características similares às relatadas pelos autores, que apresentam melhora substancial dos achados durante a evolução. Por outro lado, vemos alguns pacientes que persistem com achados de vidro fosco por bastante tempo, de forma que, muito embora o comportamento evolutivo por imagem da COVID-19 seja relativamente previsível,^(3,4)^ ainda há casos que desafiam esse padrão.

Ademais, por vezes, outros quadros se sobrepõem à infecção pelo coronavírus da síndrome respiratória aguda grave 2 (SARS-CoV-2). Além da maior incidência de tromboses venosas e arteriais^(5)^ que vem sendo descrita nesses pacientes, também temos observado quadros de coinfecção e superinfecção, algumas vezes de aspecto dramático, gerando padrões de imagem que desafiam os aspectos mais clássicos, como ilustrados não somente por nós,^(3)^ mas também por outros autores na realidade nacional.^(4)^ Não obstante, acreditamos que mereça também destaque a maior incidência de barotrauma nesses pacientes,^(6)^ outro fator que pode contribuir para o dano pulmonar. Todas essas complicações também podem influenciar na evolução por imagem das alterações de base da COVID-19 e têm potencial para deixar achados residuais no parênquima.

Dessa forma, também nos parece adequado que pacientes com COVID-19, sobretudo aqueles com doença mais grave e extensa e que apresentam complicações no curso de sua evolução, devam manter seguimento clínico, com avaliação por testes funcionais e eventuais controles tomográficos. Esse ainda é um campo em evolução e com vasto potencial de estudo.
